# Systematic Association of Genes to Phenotypes by Genome and Literature Mining

**DOI:** 10.1371/journal.pbio.0030134

**Published:** 2005-04-05

**Authors:** Jan O Korbel, Tobias Doerks, Lars J Jensen, Carolina Perez-Iratxeta, Szymon Kaczanowski, Sean D Hooper, Miguel A Andrade, Peer Bork

**Affiliations:** **1**European Molecular Biology LaboratoryHeidelbergGermany; **2**Max Delbrück Center for Molecular MedicineBerlin-BuchGermany; **3**Ontario Genomics Innovation Centre, Ottawa Health Research InstituteOttawaCanada; **4**Institute of Biochemistry and Biophysics, Polish Academy of SciencesWarsawPoland; New England BiolabsUnited States of America

## Abstract

One of the major challenges of functional genomics is to unravel the connection between genotype and phenotype. So far no global analysis has attempted to explore those connections in the light of the large phenotypic variability seen in nature. Here, we use an unsupervised, systematic approach for associating genes and phenotypic characteristics that combines literature mining with comparative genome analysis. We first mine the MEDLINE literature database for terms that reflect phenotypic similarities of species. Subsequently we predict the likely genomic determinants: genes specifically present in the respective genomes. In a global analysis involving 92 prokaryotic genomes we retrieve 323 clusters containing a total of 2,700 significant gene–phenotype associations. Some clusters contain mostly known relationships, such as genes involved in motility or plant degradation, often with additional hypothetical proteins associated with those phenotypes. Other clusters comprise unexpected associations; for example, a group of terms related to food and spoilage is linked to genes predicted to be involved in bacterial food poisoning. Among the clusters, we observe an enrichment of pathogenicity-related associations, suggesting that the approach reveals many novel genes likely to play a role in infectious diseases.

## Abstract 2

The combination of text mining and comparative genomics is shown to be a powerful approach to predicting phenotypes that are associated with particular genes in bacterial genomes

## Introduction

The universal tree of life spans a diverse set of species with distinct phenotypic characteristics, here referred to as *traits.* These include specialized lifestyles (e.g., parasitism), usage of different energy sources (e.g., sunlight), and morphological properties (e.g., motility). Identification of genotype–phenotype relationships is one of the major goals of the genomics era [1], as it may lead to the discovery of novel biochemical and cellular processes and to the molecular understanding of complex phenotypic phenomena, including diseases.

Comparative genome analysis was proposed for resolving trait–gene relationships [1,2] and has recently been used to predict genomic determinants for the well-known trait characteristics hyperthermophily [3,4], flagellar motility [4,5], and pili assembly [4]. The underlying principle is that species sharing a phenotype are likely to utilize orthologous genes in the involved biological process—thus correlations between the presence and absence of both genes and traits across species should indicate relevant genotype–phenotype associations (similarly, the co-occurrence of genes across species indicates functional links between proteins [6,7]). While the applicability of the principle was demonstrated by the case studies above [3,4,5], they require manually curated knowledge on phenotypes in particular species, which is both time-consuming and unlikely to reveal unexpected relationships in the global context of all associations between genes and phenotypes.

We have thus developed an approach that involves an unbiased large-scale search for trait-descriptive terms leading to the discovery of several novel and unanticipated gene–phenotype relationships. Using literature mining, trait characteristics of species may be retrieved directly from MEDLINE abstracts (currently, the database contains more than 12 million abstracts) by identifying words that preferentially occur in abstracts referring to particular species. We focus on traits scattered across the universal tree of life (those characteristic for subsets of distantly related species), which allow species to be grouped by phenotypic rather than phylogenetic similarity. Finally, we identify systematically associations between genes and phenotypes based on the similarity of their phyletic distribution.

## Results

### A Systematic Approach Associates Genotypes and Phenotypes

**Figure 1:**
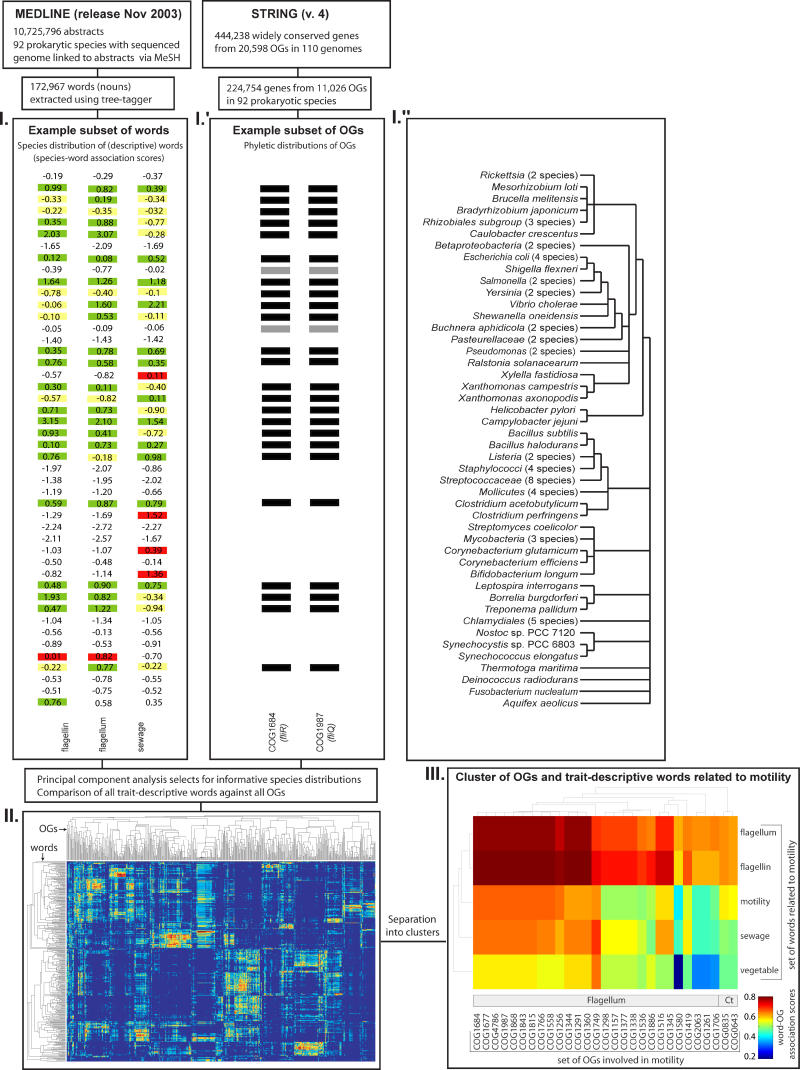
A Systematic and Unbiased Approach Combines Literature Mining and Comparative Genome Analysis with Associate Genes and Phenotypes Words likely to describe phenotypic characteristics, that is, those preferentially co-occurring with certain species, are retrieved from MEDLINE abstracts. Phyletic distributions of genes are obtained using OGs from STRING [8]. As an example, phyletic distributions across bacteria for selected words and genes are shown in (I): we show species–word association scores for the words “flagellum”, “flagellin”, and “sewage”, as well as presence/absence patterns of the selected genes *fliR* (COG1684) and *fliQ* (COG1987). Species–word association scores greater than 0 indicate that a word is likely to describe a trait of the species (colours indicate that green = true positive, i.e., the flagellar phenotype was correctly inferred; yellow = false negative; red = false positive). (I′) Black and grey bars indicate OG presence in a species (tree shown in [I′′]), while grey bars indicate presumably inactive genes [34,35]. To identify informative phyletic distributions of traits and OGs, both species–word association and species–OG occurrence vectors are transformed using PCA. The similarity of the resulting transformed and normalized word and OG vectors (i.e., the word–OG association score) is computed from their inner vector products. A “heat map” (II) shows the distribution of word–OG association scores for the more than 300 words (y-axis) and over 500 OGs (x-axis) that reveal at least one significant, high-confidence association. Dendrograms are constructed by means linkage analysis, independently applying the inner products of transformed and normalized word and OG vectors as similarities. Clusters of associated words and OGs include many previously known trait–gene relationships. For example, terms mainly related to flagellar motility form a cluster with 29 OGs known to be involved in movement; see (III). Abbreviations: Flagellum, flagellar function; Ct, involved in chemotaxis.

Historically, phenotypes are probably best understood as visible or measurable characteristics of species, which have been observed and described by biologists. The most complete resource of phenotypic knowledge is therefore the scientific literature itself. We thus identified associations between traits and genes using a multistep procedure involving genome and literature mining (see Figure 1, and Materials and Methods for a detailed description).

First, we computed associations between 172,967 nouns from MEDLINE abstracts and 92 prokaryotic *species,* for which both a complete genome sequence and a controlled vocabulary entry (Medical Subject Headings [MeSH] term) are available. Thereby, we assumed that *words* that preferentially co-occur with a subset of species are likely to be *trait-descriptive* (see for instance the examples given in Table 1).

**Table 1:**
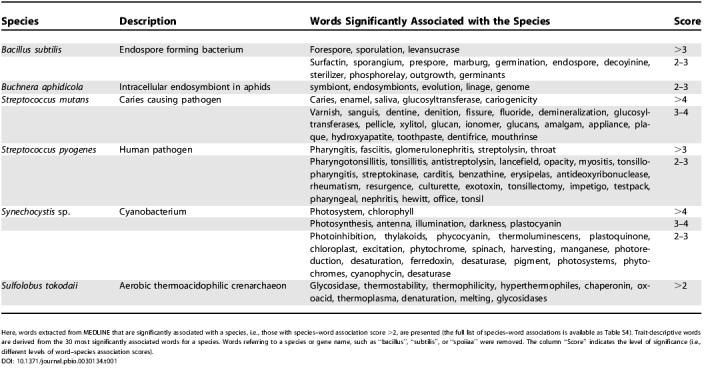
Selected Representative Prokaryotic Species with Significantly Associated Words, Assumed to Directly Relate to Phenotypic Characteristics of the Species

Here, words extracted from MEDLINE that are significantly associated with a species, i.e., those with species–word association score >2, are presented (the full list of species–word associations is available as Table S4). Trait-descriptive words are derived from the 30 most significantly associated words for a species. Words referring to a species or gene name, such as “bacillus”, “subtilis”, or “spoiiaa” were removed. The column “Score” indicates the level of significance (i.e., different levels of word–species association scores)

Second, to record the presence and absence of 224,754 genes across these 92 genomes, 11,026 orthologous groups (OGs) of genes were obtained from the Search Tool for the Retrieval of Interacting Genes/Proteins (STRING) database [8].

Third, to correct for the phyletic sampling of genome sequencing, we transformed the species association profiles of both trait-descriptive words and OGs, using principal component analysis (PCA).

**Figure 2:**
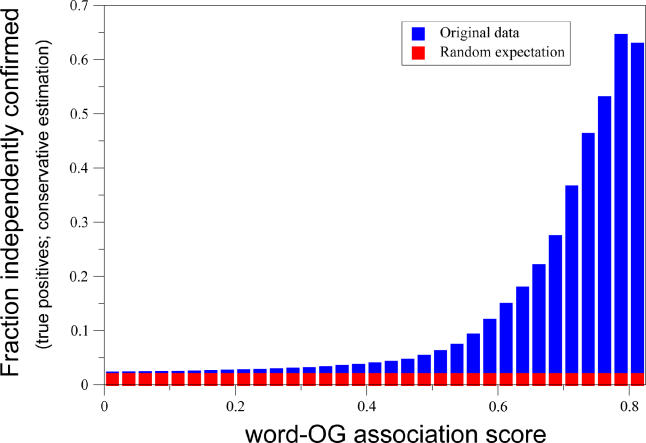
Assessment of Prediction Quality The figure demonstrates cumulative fractions of predicted OG–word associations that agree with previously known word–gene relationships (as extracted from MEDLINE). Independently confirmed predictions are enriched for high word–OG association scores.

Fourth, after comparing the resulting species–word and species–OG vectors, 2,700 significant associations between trait-descriptive words and OGs were obtained from a total of 1.9 × 10^9^ possible binary relationships. We consider as significant word–OG association scores causing at least 5-fold enrichment in true positives over expectation; this has been measured by comparing predicted associations to previously established trait–gene relationships, which were also extracted from MEDLINE (see Figure 2, and Materials and Methods).

**Figure 3:**
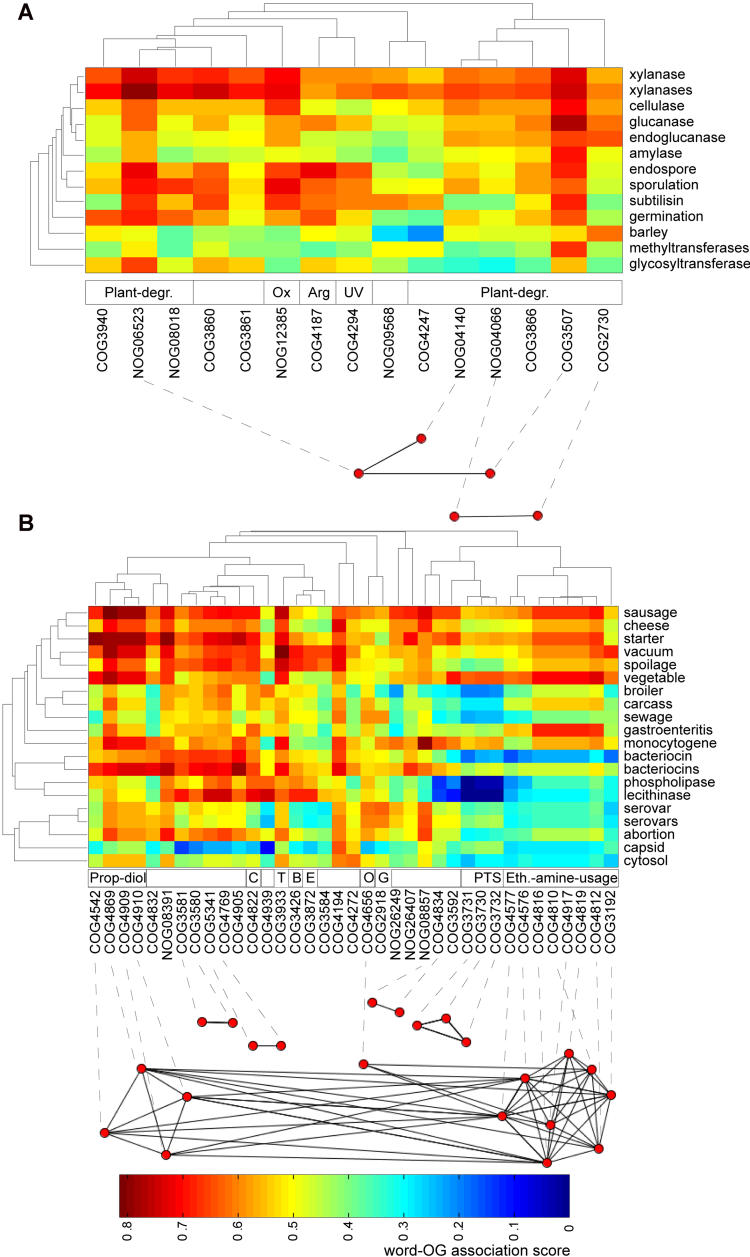
Associations between Trait-Descriptive Words and OGs for Two Illustrative Clusters “Heat maps” display word–OG association scores (scores greater than 0 are indicated; negative values are set to 0). We considered all words and OGs contributing to the respective cluster with at least one high-confidence association. Protein interaction networks, shown below, were derived from genomic context analysis (see Materials and Methods). (A) Traits and genes related to plant constituent degradation. Functional descriptions are: Plant-degr., involved in plant constituent degradation; Ox, putative oxidoreductases; Arg, Arginine degradation protein/predicted deacylase; UV, UV damage repair endonuclease; those with no description are uncharacterized. Terms related to sporulation reflect a domination of exo- and endospore-forming species from different genera (e.g., *Streptomyces, Bacillus,* and *Clostridium*) in these degradation processes. (B) Traits and genes related to food spoilage and poisoning. Some proteins have previously been implicated in virulence of food pathogens such as ManR (“T”), a transcriptional antiterminator involved in resistance to natural food preservatives, and some propanediol degradation proteins (“Prop-diol”). We suggest the involvement of additional proteins in pathogenicity: for example, ethanolamine degradation proteins (“Eth.-amine-usage”; the phospholipid phosphatidyl-ethanolamine, cleaved to ethanolamine by phospholipase, is abundant in the gut [14]); the cobalt chelatase CbiK (“C”; cobalt is an essential factor for propanediol and ethanolamine utilization [14]); a phosphotransferase system (“PTS”) involved in sorbitol transport [36] (sorbitol is an artificial food sweetener naturally found in fruits and may act as an additional carbon source; we suggest that alternatively the chemically similar inositol, cleavage product of another abundant phospholipid, may be utilized). Other proteins that may also be involved are a presumably anaerobically used butyrate kinase (“B”), gamma-glutamylcysteine synthetase (“G”), an electron transport complex protein (“O”), a predicted metal-binding enzyme (“E”), and several uncharacterized proteins (no description).

As significant associations include substantial numbers of words and OGs with similar phyletic distributions, we independently generated *sets* of both words and OGs using means linkage clustering. The resulting sets were linked if they shared at least one significant word–OG association, leading to 323 significant word–OG clusters. Visual inspection of trait–gene associations in the well-studied processes of flagellar motility and plant constituent degradation revealed that for significant word–OG association scores the vast majority of all predicted associations between the phenotype and the respective cellular processes can be justified based on previous knowledge. At a more stringent level of >7.5-fold enrichment over expectation, virtually all known trait–gene relations identified seem to agree with the clustering, and these “high-confidence” scores are analyzed here in detail (see, e.g., Figures 1 and 3, and Table 2). The entire list of significant clusters of trait–gene associations is available as Tables S1 and S2.

**Table 2:**
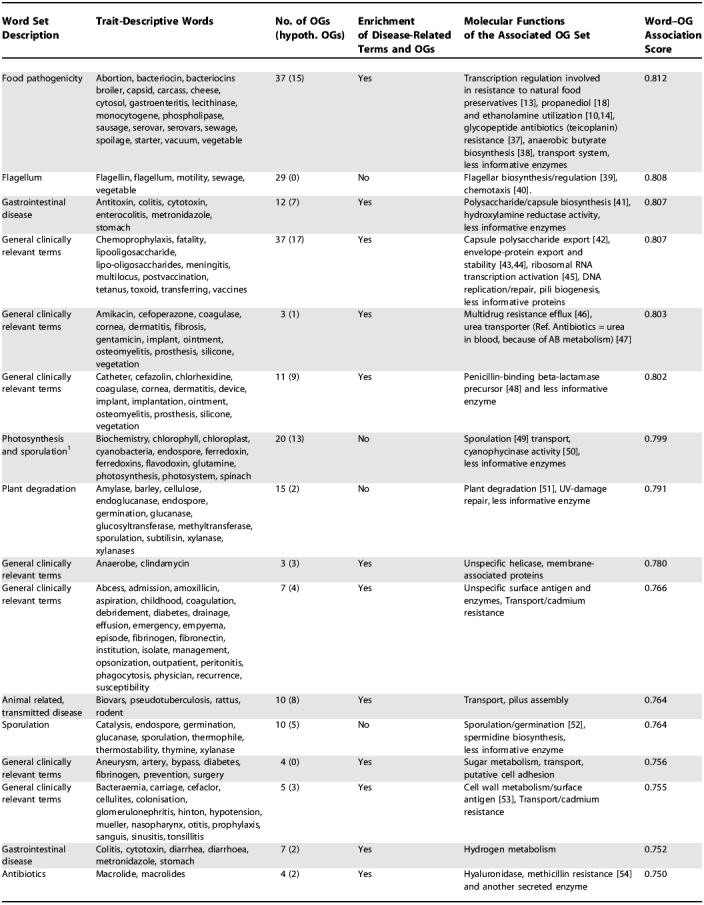
Significant Associations of Trait-Descriptive Words with Biological Functions

The table demonstrates associations of words and biological functions retrieved from the functional annotation of the respective OGs. Related trait–gene associations are shown as clusters, ranked according to the best scoring association contributing to the cluster. Only large clusters are shown (i.e., Clusters 9, 11, 12, 18, and 22–24 [see Tables S1and S2] were not considered here as they contain only one word or OG). Clusters 15 (sporulation) and 21 (hyperthermophily) were manually refined (see Tables S2 and S3). The second column indicates trait-descriptive words; the third column indicates numbers of associated OGs (OGs comprising hypothetical proteins in parentheses). We considered all words and OGs contributing to the cluster with at least one high-confidence association. The fifth column lists annotated functions of the OGs; the best word–OG association score is given in column six. The table indicates a striking enrichment of disease-related terms and OGs (see fourth column). This is most likely due to a bias in genome sequencing towards disease-causing bacteria, and a bias in MEDLINE towards disease-related topics. Furthermore, horizontal gene transfer may be particularly frequent for pathogenic species [22]

^1^ Some photosynthetic cyanobacteria may reproduce using endospores, or may form spore-like structures during nitrogen fixation

**Table 2:**
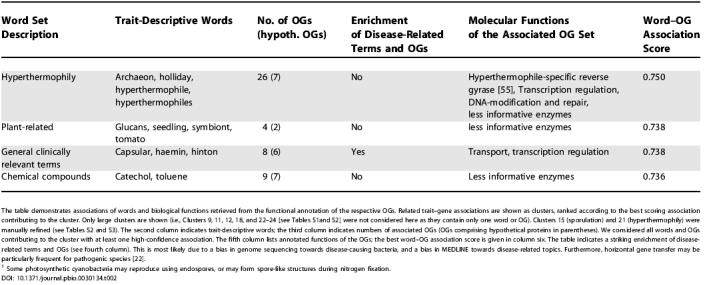
Continued

Altogether, we can infer at least one significant word–OG association for 811 OGs, corresponding to 28,888 genes. While many of these correspond to previously known trait–gene relationships, numerous relationships are novel or of unexpected character and complexity.

### Identification of Previously Known Trait–Gene Associations

Among the many previously known trait–gene relationships are genes involved in pathogenicity, the degradation of plant constituents, capsule biosynthesis, and photosynthesis (see Table 2). Furthermore, determinants for flagellar motility and hyperthermophily, the target for previous case studies [3,4,5], were identified (see Tables 3 and S3). For example, 72% of all genes involved in synthesizing or maintaining the bacterial flagellum, according to *Kyoto Encyclopedia of Genes and Genomes* (KEGG [9]), are recovered with high-confidence scores (see Figure 1 and Table 3).

**Table 3:**
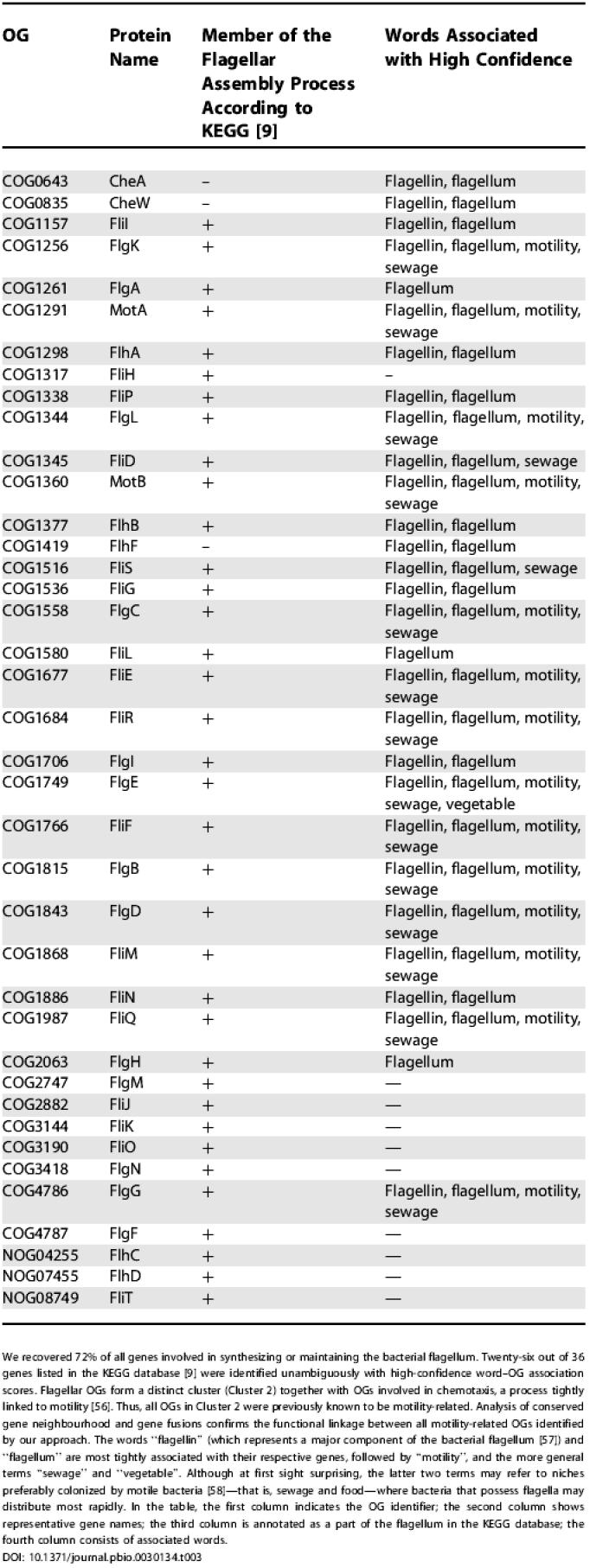
OGs and Terms Associated with Motility (Cluster 2)

We recovered 72% of all genes involved in synthesizing or maintaining the bacterial flagellum. Twenty-six out of 36 genes listed in the KEGG database [9] were identified unambiguously with high-confidence word–OG association scores. Flagellar OGs form a distinct cluster (Cluster 2) together with OGs involved in chemotaxis, a process tightly linked to motility [56]. Thus, all OGs in Cluster 2 were previously known to be motility-related. Analysis of conserved gene neighbourhood and gene fusions confirms the functional linkage between all motility-related OGs identified by our approach. The words “flagellin” (which represents a major component of the bacterial flagellum [57]) and “flagellum” are most tightly associated with their respective genes, followed by “motility”, and the more general terms “sewage” and “vegetable”. Although at first sight surprising, the latter two terms may refer to niches preferably colonized by motile bacteria [58]—that is, sewage and food—where bacteria that possess flagella may distribute most rapidly. In the table, the first column indicates the OG identifier; the second column shows representative gene names; the third column is annotated as a part of the flagellum in the KEGG database; the fourth column consists of associated words

### Prediction of Novel Genes in Known Processes: Enzymes Involved in Plant Degradation

We can predict trait relationships for many hypothetical genes. Within the 20 best scoring clusters (all of high-confidence trait–gene associations), 113 uncharacterized OGs (i.e., no functional annotations are given in SWISSPROT, TREMBL, or the Clusters of Orthologous Groups [COGs] database) are linked to trait-descriptive words (see Tables 2 and S1). For example, “hypothetical” and poorly characterized OGs are predicted to encode enzymes involved in the biodegradation of plant constituents (Figure 3A); altogether 13 words, mainly describing reactions that break down plant polysaccharides, form a set with 15, mostly enzyme-encoding, OGs. Out of the latter, 75% (i.e., nine of the 12 OGs that have at least provisional functional annotations) are already known, or have been suggested, to break down plant polysaccharides (see Tables S1 and S2). We predict involvement in plant degradation also for the remaining OGs, which include three uncharacterized OGs, and two with unspecific functional annotation (nonsupervised orthologous group [NOG]12385 is a “putative oxidoreductase”; COG4187 members are annotated as “Arginine degradation protein” and “predicted deacylase”).

### Prediction of Unexpected Associations: Novel Genomic Determinants for Food Poisoning?

**Figure 4:**
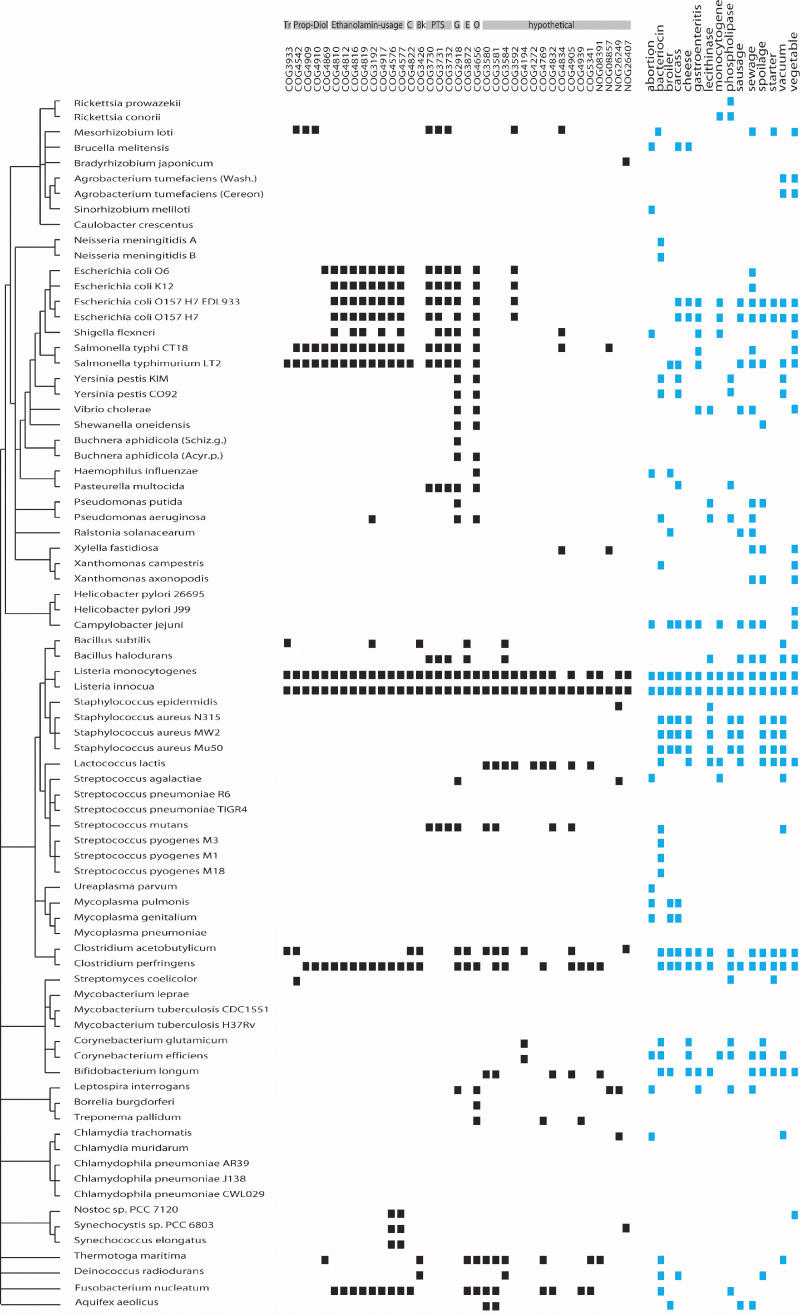
Phyletic Distributions across Bacteria of Genes and Associated Representative Trait-Descriptive Words Related to Food, Food Spoilage, and Food Poisoning (Cluster 1) The complete figure, including phylogenetic distributions of all trait-descriptive words and OGs in Cluster 1, is available online as Figure S1. Black squares indicate gene occurrences across species for the respective OGs. Blue squares indicate predicted associations between trait-descriptive words and species (species–word association scores greater than 0). Function descriptions (grey bar) are the same as in Figure 3.

Of the many unanticipated trait–gene relationships, we will discuss in detail a cluster of word–OG associations that groups several terms related to food and food poisoning with a number of OGs encoding metabolic enzymes (Figures 3B and 4). The words of the respective set refer, for instance, to common habitats of food pathogens, such as processed and spoiled food (e.g., “cheese”, “sausage”, “broiler”, “spoilage”, and “carcass”), and to natural food preservatives used to inhibit their growth (“bacteriocin”). Furthermore, several specific pathogenicity-related terms were revealed: for example, “monocytogene”, “gastroenteritis”, and “abortion” that refer to immune response, direct and indirect results of intestinal infections, respectively [10]. Also the terms “phospholipase” and “lecithinase” in this word set have been implicated in toxicity mechanisms [11,12], despite the general cellular roles of the proteins. Other words may refer to unspecific connotations of more general words, which play an important role in clinical praxis or the food industry, such as “starter” (culture) and “vacuum” (packaging). We predict for 37 OGs with high-confidence association to these words that they are involved in food spoilage and toxicity. All of these OGs are present in food-borne pathogens, and absent from most other prokaryotes. One of them has already been demonstrated experimentally to be involved in food spoilage and pathogenicity: the *manR* gene (COG3933) of the food pathogen Listeria monocytogenes encodes a transcriptional regulator that was shown to be involved in resistance to natural food preservatives [13].

Of the remaining 36 OGs, four are components of the utilization pathway for 1,2-propanediol and eight participate in ethanolamine usage. Among the remaining, mostly poorly characterized OGs, one is a cobalt chelatase likely to be involved in providing the essential cobalamin for both pathways [14]. Intriguingly, propanediol and ethanolamine are abundant compounds in the human gut [14,15] and in processed food [16,17] that can be utilized as the main source of carbon, nitrogen, and energy under aerobic and anaerobic conditions [10,14]. The corresponding genes form conserved operons in three of the most hazardous food-borne pathogens—L. monocytogenes (a low-GC Gram-positive bacterium, *Bacillales* family), Clostridium perfringens (high-GC Gram-positive), and Salmonella typhimurium (Gram-negative)—but are absent from almost all other species. Further sequence analysis in National Center for Biotechnology Information's NRDB revealed the presence of ethanolamine usage genes in the recently sequenced species Enterococcus faecalis—another gut pathogen from a distinct phylogenetic clade *(Lactobacillales).* Based on these associations we predict that both propanediol and ethanolamine utilization pathways are crucial genomic determinants of pathogenicity associated with food poisoning, presumably by promoting anaerobic growth both in the host and in processed food. Thereby, they may provide a growth advantage over natural gut inhabitants not possessing those genes (Figure 4). In agreement with these predictions, genes involved in propanediol utilization were previously proposed to be involved in pathogenicity of S. typhimurium as deletion of the respective genes specifically impairs growth in the host [18].

## Discussion

We present here a computational approach that combines literature data mining with comparative genome analysis to systematically identify numerous novel phenotype–genotype relationships. Text-mining methods have previously been used for function prediction, for example, to associate functionally interacting proteins (e.g., [19,20]) and to link human genes to hereditary diseases (e.g., [21]). However, this is, to the best of our knowledge, the first unbiased and systematic approach that enables the identification of genomic determinants for numerous phenotypes, without requiring manually curated knowledge on phenotypic characteristics.

Besides identifying several previously known relationships, our approach predicts many novel and unanticipated trait–gene associations with high confidence (Figures 3 and 4 and Table 2). The association of food- and spoilage-related terms with genes presumably involved in food intoxication exemplifies the identification of unforeseen relationships using an unsupervised strategy. A typical example is the significant association of “cheese”, “abortion”, and predicted pathogenicity factors, presumably explained by *Listeria* infections following raw-milk cheese consumption, which may cause miscarriage.

Given the focus of research and, in particular, genome sequencing on tackling human health issues, it is not surprising that a considerable proportion of all inferred genes are predicted to be involved in bacterial pathogenicity (Table 2). It was furthermore proposed that genes responsible for virulence are frequently transferred horizontally across distant clades [22], which would favour them in our analysis. These arguments imply that our approach represents a well-suited tool for identifying disease-related genes that may serve as promising novel classes of drug targets.

Altogether, we have identified 2,700 significant OG–word associations, which link more than 800 OGs (encoding over 28,000 proteins) to at least one trait-descriptive word. Our approach to function prediction is complementary to computational methods that utilize evolutionary signals, such as genomic context methods that analyze conserved gene neighbourhood [23,24,25], or gene fusion [26,27], to predict functional associations in terms of involvement in a common cellular process. Notably, the majority of poorly characterized OGs we identify cannot be functionally annotated by these methods (see Figure 3 and Materials and Methods). Furthermore, even if genomic context methods link to a set of OGs, our approach can associate these sets to phenotypic characteristics. For example, while genes involved in ethanolamine utilization can be inferred using existing genome context methods, our approach enables linking this cellular process with food-borne pathogenicity. Furthermore, grouping of ethanolamine utilization with the food preservative resistance factor *manR* and other genes indicates that independent cellular processes can be combined by the approach, if they are involved in the same trait.

As more genomes are sequenced and the available literature in MEDLINE is constantly increasing in size, our approach is expected to predict more fine-grained trait–gene relationships in the future. This may pave the way for mapping numerous distinctive phenotypic characteristics observed in nature to the genes responsible.

## Materials and Methods

### 

#### Identification of species–word associations

We first identified the distribution of phenotypic traits across species by analyzing the co-mentioning of species and trait-descriptive words across MEDLINE abstracts. Namely, nouns that preferentially co-occur with a subset of species are likely to be trait-descriptive (e.g., the words “flagellum” and “motility” are enriched in abstracts dealing with motile species; see Figure 1). We focused on nouns because these presumably carry a more considerable proportion of the relevant information represented in the scientific literature than verbs and adjectives [28]. Nouns were extracted from MEDLINE abstracts using a part-of-speech tagger (i.e., Tree Tagger [29]). Words of five characters or less were excluded from the analysis, as many of those are gene names and other noninformative words leading to an increase in noise. Species names were taken from the corresponding MeSH terms associated with the abstracts, that is, from the MeSH B category corresponding to “organisms” (applying the controlled MeSH vocabulary reduces errors in species name recognition—for example, in the case of synonym usage; on the other hand, using all nouns in MEDLINE abstracts for identifying trait-descriptive words allows searching a variety of traits not accessible via a controlled vocabulary). Some species were not represented in MeSH, and were thus mapped to their genus. A total of 255,249 MEDLINE abstracts connected with any of the 92 species analyzed were considered in the analysis. We considered the occurrence of distinct species in abstracts. Frequencies of words within abstracts were not taken into account (single and multiple occurrences were equally treated as “word presence”). Given the set of *n*_1_ words and *n*_2_ species associated with an abstract, we counted all possible species–word pairs *(n*_1*×*_ *n*_2_ *).* For each species–word pair, a species–word association score *s_sw_* was determined using a regularized log-odds score:



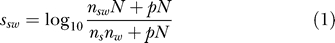



where *n_s_* is the number of abstracts mentioning a particular species, *n_w_* refers to the number of abstracts mentioning a particular word, *n_sw_* is the number of abstracts that co-mention species and word, and *N* is the sum of all *n_sw_*. The log-odds framework quantifies correlation strengths and, in particular, facilitates the handling of species or words for which only sparse scientific literature exists. To allow the handling of sparse data, the standard log-odds formula was augmented with pseudocounts, *p* = 1. The resulting score, *s_sw_,* yields positive values for enriched species–word pairs and negative values for underrepresented pairs. The magnitude of the score provides a measure of the strength of the association, indicating its potential relevance in describing a characteristic trait. To record overrepresentation, the species–word association score requires frequently used words and species (such as “flagellum” and Escherichia coli) to be co-mentioned more often than infrequent ones (e.g., “oligosaccharide” and Ralstonia solanacearum).

Associations were calculated for each species–word pair, and a species association vector was subsequently constructed for each word, representing its association scores with each of the 92 prokaryotic species studied.

#### Identification of orthologues and species–gene associations

We obtained groups of OGs covering those 92 completely sequenced genomes from the STRING server [8], version 4 (the raw data can be downloaded from http://string.embl.de). The OGs include protein families originally obtained from the COG database [30], which were subsequently expanded and extended to accommodate more recently sequenced species [8]. Species–OG association vectors were constructed for each OG; +1 signifies presence of a gene (i.e., orthologue) in a certain species, while −1 signifies absence.

#### PCA

To reduce the sampling bias introduced by genome sequencing projects, which have been focused on certain groups of closely related species, we performed PCA (also known as singular value decomposition) on the species–OG association matrix. The PCA transformation collapsed groups of species with very similar gene content to a single dimension, thereby eliminating inherent correlations from our representation. Subsequently, the same linear transformation was applied to the species–word association vectors. For both species–OG and species–word association vectors, the first 32 principal components were further considered, yielding an acceptable signal-to-noise ratio. The performance of the approach was comparable (i.e., slightly weaker) when applying distinct numbers of components in the range from approximately 25 to 40. Considering even smaller or larger numbers of components led to performance drops, as too little information or too much noise was included in the further analysis.

#### Mapping genes to phenotypes and vice versa

Before mapping genes and traits, further filtering was applied to diminish the contribution of rarely occurring OGs and words; that is, only OGs occurring in at least four distantly related species clades were considered; similarly, we focused on words yielding positive species–word associations in at least four distantly related species clades (thereby utilizing clades of closely related species from STRING [8]; see Table S1 in [25]). OGs encoding phage-associated proteins (i.e., those with description lines including the terms “phage”, “transposase”, and “integrase”) were regarded as a source of “contamination” within the genomes of analysed species and thus ignored. Finally, we eliminated words that did not display sufficiently strong association with any of the species studied; this was done by removing all but the 1,000 longest transformed species–word vectors (these were considered to be the most informative vectors). The remaining vectors were normalized to a length of 1; similarly, all species–OG vectors were normalized. Subsequently, the pair-wise similarity of each word–OG pair was computed, that is, the word–OG association score, defined as the inner product of normalized species–OG and species–word vectors (the highest word–OG association score obtained is 0.812; see Table 2). Furthermore, the similarity score for pairs of OGs and for pairs of words was computed in the same way as described for the word–OG associations. Using means linkage clustering analysis as implemented in OC [31] (“similarity mode”; cutoff = 0.45), sets of words and OGs were independently generated. Finally, combined clusters were constructed by combining word sets with OG sets, if these were linked by at least one significant word–OG association. Note that this “loose” clustering procedure allows word sets to be combined with several OG sets, and vice versa (i.e., words or OGs may in principle be part of several clusters).

#### Assessment of prediction quality

We reasoned that the quality of the predictions may be examined using an orthogonal strategy—comparing the predicted word–OG associations to previously established trait–gene relationships, which can be extracted from MEDLINE when focusing on significantly associated word–gene pairs. Namely, previously established relations were extracted from scientific abstracts, by detecting significant co-mentioning of trait-descriptive words and gene names, using the hypergeometric distribution [32,33]. Thereby, gene names were associated to species, considering MeSH terms and organism names occurring in abstracts, and including gene synonyms retrieved from http://www.bork.embl-heidelberg.de/synonyms. (For instance, the word “flagellum” co-occurs significantly with *fliR* from *S. typhimurium, p* = 0.00063, consistent with a gene function in motility). We assume confirmation of a predicted word–OG association *(“true positive”),* if any gene within an OG significantly co-occurs with a word (i.e., when *p* ≤ 1/10^1.5^, roughly corresponding to *p* ≤ 0.03). Figure 2 demonstrates the enrichment of true positives among the highest scoring predictions. Words not significantly associated with any gene, or OGs lacking genes significantly linked to any word were ignored. Furthermore, we conservatively estimated expected fractions of true positives by shuffling both OGs and the 1,000 most informative words, and subsequently repeating the assessment with previously established trait–gene relationships on these randomized associations. By comparing to expected scores, we estimated two significance thresholds: word–OG association scores ≥0.5675 (true positives are 5-fold enriched over expectation) are regarded as significant; scores ≥0.6125 (7.5-fold enrichment) indicate “high-confidence”. OGs and words discussed in detail (see, e.g., Figures 1, 3, and 4 and Tables 2 and 3) all contribute with at least one high-confidence association to the respective clusters.

#### Construction of genomic context association networks

We tested whether functional annotations predicted from our method can also be inferred with existing computational methods (which utilize different methodologies than the approach described here). We compared predictions from our approach to functional associations between proteins inferred from genomic context methods (see, e.g., Figure 3), which predict involvement in a common metabolic pathway or biochemical process. Thereby, we considered OGs to be functionally characterized by another method, if the corresponding genes can be significantly associated to genes of known function, that is, if they are fused to such genes [26,27], or if they occur in conserved proximity with these (we analyzed conserved organization of putative operons [23,24], and of divergently transcribed gene pairs [25]; gene fusions and conserved [putative] operon structures were examined using STRING [8] with the default probability cutoff of 0.400, excluding evidence from sources other than gene fusion or neighbourhood; for the divergently transcribed gene pair method, all pairs conserved across at least three distant evolutionary species clades were considered [25]).

#### Species analyzed

The following species were analyzed:

*Aeropyrum pernix, Agrobacterium tumefaciens* (Cereon), A. tumefaciens (Wash.), *Aquifex aeolicus, Archaeoglobus fulgidus, Bacillus halodurans, B. subtilis, Bifidobacterium longum, Borrelia burgdorferi, Bradyrhizobium japonicum, Brucella melitensis, Buchnera aphidicola, B. aphidicola* Schiz*, Campylobacter jejuni, Caulobacter crescentus, Chlamydia muridarum, C. trachomatis, Chlamydophila pneumoniae* AR39, C. pneumoniae CWL029, C. pneumoniae J138, *Clostridium acetobutylicum, C. perfringens, Corynebacterium efficiens, C. glutamicum, Deinococcus radiodurans, Escherichia coli* K12, E. coli O157:H7, E. coli O157:H7 EDL933, E. coli O6, *Fusobacterium nucleatum, Haemophilus influenzae, Halobacterium* sp. NRC-1, Helicobacter pylori 26695, *Lactococcus lactis, Leptospira interrogans, Listeria innocua, L. monocytogenes, Mesorhizobium loti, Methanococcus jannaschii, Methanosarcina acetivorans, M. mazei, Mycobacterium leprae, M. tuberculosis* CDC1551, M. tuberculosis H37Rv, *Mycoplasma genitalium, M. pneumoniae, M. pulmonis, Neisseria meningitidis* A, N. meningitidis B, *Nostoc* sp. PCC 7120, *Pasteurella multocida, Pseudomonas aeruginosa, P. putida, Pyrobaculum aerophilum, Pyrococcus abyssi, P. furiosus, P. horikoshii, Ralstonia solanacearum, Rickettsia conorii, R. prowazekii, Salmonella typhi, S. typhimurium, Shewanella oneidensis, Shigella flexneri, Sinorhizobium meliloti, Streptococcus agalactiae, S. mutans, S. pneumoniae* R6, S. pneumoniae TIGR4, *S. pyogenes, S. pyogenes* M3, S. pyogenes MGAS8232, Staphylococcus aureus Mu50, S. aureus MW2, S. aureus N315, *S. epidermidis, Streptomyces coelicolor, Sulfolobus solfataricus, S. tokodaii, Synechococcus elongatus, Synechocystis* sp. PCC 6803, *Thermoplasma acidophilum, T. volcanium, Thermotoga maritima, Treponema pallidum, Ureaplasma parvum, Vibrio cholerae, Xanthomonas axonopodis, X. campestris, Xylella fastidiosa, Yersinia pestis, and Y. pestis* KIM*.*

## Supporting Information

Figure S1 Genes and Trait-Descriptive Words Related to Food Poisoning Phyletic distributions across bacteria of genes and associated trait-descriptive words related to food, food spoilage, and food poisoning (complete figure).
(763 KB JPG).

Table S1 Complete List of Trait-OG Clusters Based on All Significant Associations (221 KB TXT).

Table S2 Complete List of Trait-OG Clusters Based on High-Confidence Associations Only The clusters have been refined as described in Table S3.
(116 KB TXT).

Table S3 OGs Predicted to Be Involved in Hyperthermophily (52 KB DOC).

Table S4 Complete List of Species–Word Associations Identified by Mining MEDLINE (10.3 MB ZIP).

## Abbreviations

COGs: Clusters of Orthologous Groups
KEGG: *Kyoto Encyclopedia of Genes and Genomes*
MeSH: Medical Subject Headings
NOG: nonsupervised orthologous group
OG: orthologous group
PCA: principal component analysis
STRING: Search Tool for the Retrieval of Interacting Genes/Proteins
