# Supplementary material for: Systematic Association of Genes to Phenotypes by Genome and Literature Mining
Source: PLoS Biol. 2005 Apr 5;3(5):e134. doi: 10.1371/journal.pbio.0030134 (PMC1073694; doi:10.1371/journal.pbio.0030134)
Supplement: Supplementary file 2 [file pbio.0030134.st003.doc]

**Table S3.** OGs predicted to be involved in hyperthermophily (Cluster 21). Our approach can also identify genes that have previously been predicted to be involved in the hyperthermophilic lifestyle [1]. Predicted relevant associations involving thermophily-related terms (such as ‘thermostability’ and ‘thermophile’; e.g. the latter is predicted to be linked to the poorly characterized orthologous groups COG1728, COG1582, NOG14705, COG4200 with high confidence), led us to add the terms ‘hyperthermophile’ and ‘hyperthermophiles’ to the pipeline. (Both terms were initially filtered due to low information content; see **Materials and Methods**. Similarly, the terms ‘sporulation’ and ‘endospore’ were added, after observing sporulation-related terms such as ‘stearothermophilus’, leading to a refined Cluster 15.) After the refinement, several OGs that have previously been suggested to be involved in the hyperthermophilic lifestyle, as well as novel OGs were retrieved in a distinct cluster. First column: OG-identifier; second column: functional description; third column: recent prediction in Makarova *et al*. [1]; fourth column: keywords associated with high confidence.

| **Orthologous group (OG)** | **Description** | **Predicted by Makarova *et al.*** | **Keywords associated with high confidence** |
| --- | --- | --- | --- |
| COG1110 | Reverse gyrase | + | hyperthermophile, hyperthermophiles |
| COG1318 | Predicted transcriptional regulators | + | hyperthermophile |
| COG1468 | RecB family exonuclease | + | hyperthermophiles |
| COG1483 | Predicted ATPase  (AAA+ superfamily) | - | hyperthermophile |
| COG1517 | Uncharacterized protein predicted to be involved in DNA repair | - | hyperthermophile, hyperthermophiles |
| COG1688 | Uncharacterized protein predicted to be involved in DNA repair | + | hyperthermophile, hyperthermophiles |
| COG1743 | Adenine-specific methylase containing a Zn-ribbon | - | hyperthermophile |
| COG1856 | Uncharacterized homolog of biotin synthetase | + | hyperthermophile, hyperthermophiles |
| COG1857 | Uncharacterized protein predicted to be involved in DNA repair | - | hyperthermophiles |
| COG1906 | Uncharacterized conserved protein | + | hyperthermophile |
| COG2100 | Predicted Fe-S oxidoreductase | - | hyperthermophile |
| COG2112 | Predicted Ser/Thr protein kinase | - | hyperthermophile, hyperthermophiles |
| COG2245 | Predicted membrane protein | - | hyperthermophile |
| COG2250 | Uncharacterized conserved protein related to C-terminal domain of eukaryotic chaperone, SACSIN | + | hyperthermophile, hyperthermophiles |
| COG2254 | Predicted HD superfamily hydrolase, possibly a nuclease | - | hyperthermophile, hyperthermophiles |
| COG2411 | Uncharacterized conserved protein | - | hyperthermophile |
| COG2413 | Predicted nucleotidyltransferase | - | hyperthermophile, hyperthermophiles |
| COG2419 | Uncharacterized conserved protein | - | hyperthermophile, hyperthermophiles |
| COG2441 | Predicted butyrate kinase | - | hyperthermophiles |
| COG2516 | Biotin synthase-related enzyme | + | hyperthermophile, hyperthermophiles |
| COG3889 | Predicted solute binding protein | - | hyperthermophile, hyperthermophiles |
| COG4047 | Uncharacterized protein conserved in archaea | - | hyperthermophile |
| COG4345 | Uncharacterized protein conserved in archaea | - | hyperthermophiles |
| COG4346 | Predicted membrane-bound dolichyl-phosphate-mannose-protein | - | hyperthermophiles |
| NOG07117 | Non supervised orthologous group, uncharacterized | - | hyperthermophiles |

**References of the Supporting Information**

1. Makarova KS, Wolf YI, Koonin EV (2003) Potential genomic determinants of hyperthermophily. Trends Genet 19: 172-176.
